# High-Fat Feeding Improves Anxiety-Type Behavior Induced by Ovariectomy in Rats

**DOI:** 10.3389/fnins.2018.00557

**Published:** 2018-09-03

**Authors:** Ana P. S. Dornellas, Valter T. Boldarine, Amanda P. Pedroso, Lorenza O. T. Carvalho, Iracema S. de Andrade, Tânia M. Vulcani-Freitas, Carla C. C. dos Santos, Cláudia M. da Penha Oller do Nascimento, Lila M. Oyama, Eliane B. Ribeiro

**Affiliations:** Physiology Department, Escola Paulista de Medicina, Universidade Federal de São Paulo, São Paulo, Brazil

**Keywords:** hypothalamus, hippocampus, serotonin, lard, fish-oil, neuropeptides

## Abstract

Menopause-induced changes may include increased incidence of both depression/anxiety and obesity. We hypothesized that behavioral changes that may develop after ovarian failure could be related to neurochemical and metabolic aspects affected by this condition and that high-fat intake may influence these associations. The present study investigated in rats the effects of ovariectomy, either alone or combined with high-fat diets enriched with either lard or fish-oil, on metabolic, behavioral and monoaminergic statuses, and on gene expression of neuropeptides and receptors involved in energy balance and mood regulation. Female rats had their ovaries removed and received either standard chow (OvxC) or high-fat diets enriched with either lard (OvxL) or fish-oil (OvxF) for 8 weeks. The Sham group received only chow diet. Ovariectomy increased feed efficiency and body weight gain and impaired glucose homeostasis and serotonin-induced hypophagia, effects either maintained or even accentuated by the lard diet but counteracted by the fish diet. The OvxL group developed obesity and hyperleptinemia. Regarding components of hypothalamic serotonergic system, both ovariectomy alone or combined with the fish diet increased 5-HT_2C_ expression while the lard diet reduced 5-HT_1B_ mRNA. Ovariectomy increased the anxiety index, as derived from the elevated plus maze test, while both high-fat groups showed normalization of this index. In the forced swimming test, ovariectomy allied to high-lard diet, but not to fish-oil diet, reduced the latency to immobility, indicating vulnerability to a depressive-like state. Linear regression analysis showed hippocampal AgRP to be negatively associated with the anxiety index and hypothalamic AgRP to be positively associated with the latency to immobility. These AgRp gene expression associations are indicative of a beneficial involvement of this neuropeptide on both depression and anxiety measures. The present findings demonstrate metabolic, neurochemical and behavioral alterations after ovaries removal and highlight a positive effect of high-fat feeding on the anxiety-like behavior shown by ovariectomized animals. Since the polyunsaturated ômega-3 intake (fish diet), unlike the saturated fat intake (lard diet), failed to induce deleterious metabolic or neurochemical consequences, further studies are needed focusing on the potential of this dietary component as an adjuvant anxiolytic agent after menopause.

## Introduction

Although a connection between metabolic disturbances and psychiatric disorders has been suggested, the mechanisms involved have not been elucidated. Patients with depression have been shown to present inadequate patterns of food intake and glycemic control while diabetic individuals had higher incidence of cognitive impairment, depression and anxiety disorders (Panza et al., 2012; Correia and Ravasco, 2014; Singh, 2014; Barandas et al., 2015).

The proportion of overweight and obese adults has increased worldwide regardless of gender (Ng et al., 2014; de la Iglesia et al., 2016), while the prevalence of anxiety disorders and depression are higher in women than in men (Bromberger and Kravitz, 2011; Kessler et al., 2015). Gender differences related to depression seem to emerge after puberty (Hankin et al., 2007) and to decline years after menopause (Bebbington et al., 2003). Moreover, there is an increased prevalence of mood disorders in post-menopausal women, a condition reportedly aggravated by the presence of diabetes (Anderson et al., 2001; Martins et al., 2002; Kim et al., 2015). Additionally, the association between obesity and depression is highly expressed after menopause but the mechanisms involved are unknown (Perquier et al., 2014; Xiong et al., 2017).

Energy balance is regulated by a complex system in which the hypothalamus plays a pivotal role, integrating signals from both peripheral and central sites to modulate the release of neuropeptides controlling food intake and energy expenditure (Gerozissis, 2008; Yeo and Heisler, 2012). In contrast, the hippocampus is an area highly involved in the control of mood and cognition (Mahar et al., 2014; Anacker and Hen, 2017). Interestingly, it is targeted by peripherally-derived endocrine signs and it has been suggested to participate in the non-homeostatic control of feeding through regulating the activation of reward memory by food-related cues (Davidson et al., 2005). However, the actions of these hormonal signs on this brain area remain little explored (Kanoski and Grill, 2017).

Estrogen deficiency has been associated with increased body adiposity, impairment of plasma lipid profile, type 2 diabetes and metabolic syndrome (Milewicz et al., 1996; Carr, 2003; Messina et al., 2013). Estradiol has been shown to inhibit feeding via hypothalamic actions. The reported effects of estradiol are complex and include attenuation of ghrelin's orexigenic action (Ferrer-Lorente et al., 2009), inhibition of neuropeptide Y (NPY)/agouti-related peptide (AgRP) orexigenic neurons (Olofsson et al., 2009), potentiation of cholecystokinin-induced satiety (Asarian and Geary, 2007), and stimulation of pro-opiomelanocortin (POMC) anorexigenic neurons (Zhu et al., 2015). Estradiol-induced hypophagia may also involve hypothalamic serotonergic activation (Pelletier et al., 2007; Silva et al., 2010; Rivera et al., 2012; Santollo et al., 2012). Moreover, estrogens influence brain areas regulating mood and cognition, such as the hippocampus, increasing the availability of monoamines through reduction of monoamine oxidase expression, stimulation of tryptophan hydroxylase, and regulation of serotonin (5-HT) neuronal transport (Ren-Patterson et al., 2006; Kiss et al., 2012). These data indicate that estrogen deficiency after menopause could facilitate the establishment of both mood disorders and metabolic derangements (Alexander et al., 2007).

The role played by AgRP, NPY, POMC, and CART acting at the hippocampus is not yet understood. Hippocampal infusion of NPY reversed the anxiogenic action of corticotropin releasing factor (Kask et al., 2002; Heilig, 2004) and chronic restraint stress elevated CART gene expression in the hippocampus (Hunter et al., 2007). POMC deficiency decreased hippocampal cell proliferation in rats (Ostwald et al., 2006) while AgRP expression has been associated with hippocampal maturation in human fetal cells (Bai et al., 2005).

Dietary factors may have an impact on both metabolic and mood disorders. We have previously shown, in rats, that fish-oil intake increased neuronal activation of hypothalamic sites expressing anorexigenic mediators (Watanabe et al., 2009). Moreover, n-3 PUFAs presented a protective effect in patients with major depression, acting via serotonergic modulation (Peet and Stokes, 2005; Ross et al., 2007). In mice, the consumption of n-3 diet had an antidepressant-like effect, and increased hippocampal volume, BDNF expression and neurogenesis (Venna et al., 2009). In contrast, despite the obesogenic effect of saturated fatty acids, lard consumption reportedly reduced signs of stress and anxiety in humans and animals (Finger et al., 2011; Singh, 2014), but little is known about its properties in an estrogen deficiency-state.

Based on the above considerations, the present study investigated the effects of ovaries removal, either alone or combined with the chronic consumption of high-fat diets enriched with either lard or fish-oil, on metabolic, behavioral and monoaminergic statuses. Additionally, hypothalamic and hippocampal gene expression of neuropeptides and receptors involved in energy balance and mood regulation have also been analyzed. We hypothesized that behavioral changes that may develop in the absence of ovarian hormones could be related to neurochemical and metabolic aspects affected by this condition and that high-fat intake may impact these associations.

## Methods

### Animals and diets

All the procedures were in agreement with the guidelines of the Committee in Research Ethics of the Universidade Federal de São Paulo (CEP 0311/11). Eight-week-old female *Wistar* rats were weighed and submitted to either bilateral ovariectomy or sham operation, under ketamine/xylazine anesthesia (66/33 mg/kg, ip). After the surgery, they were housed four to five per cage and maintained under controlled lighting (12 h light/dark cycle, lights on at 6 a.m.) and temperature conditions (23 ± 1°C), with free access to food and water. The ovariectomized rats (Ovx) were assigned to one of three groups (OvxC, OvxL, or OvxF), according to the diet received for the next 8 weeks. The OvxC group received standard rat chow (Control diet, 2.87 kcal/g, 15% of energy from fat, Nuvilab, Brazil) while the OvxL and OvxF groups received high-fat diets (3.60 kcal/g, 45% energy from fat) enriched with either lard (Aurora, Cooperativa Central Aurora de Alimentos, Brazil) or fish oil (ROPUFA® “75” ω-3, Roche, DSM Nutritional Products, Brazil), respectively. The sham-ovariectomized group (Sham) received the control diet.

The high-fat diets were prepared by adding, to the standard chow, 20% (w/w) fat, 20% (w/w) casein, 10% (w/w) sucrose, and 0.02% (w/w) butylated hydroxytoluene (Watanabe et al., 2010; Dornellas et al., 2015). The lard diet contained 2% soybean oil to ensure the adequate content of essential PUFAs (Reeves, 1997).

### Food intake, body and fat pads weight, and serum parameters

Food intake and body weight were measured weekly. The metabolic efficiency was calculated as [(body weight gain/energy intake)^*^100]. At the end of the diet treatment, the rats were euthanized after an overnight fast, trunk blood was collected and serum stored at −80°C. Uterus and retroperitoneal, gonadal and mesenteric fat pads were dissected and weighed. Ovariectomy was confirmed by uterus atrophy. Glucose, triacylglycerols and cholesterol levels were analyzed by enzymatic methods (Labtest Diagnóstica, Brazil). Serum levels of insulin and leptin were measured by enzyme-linked immunosorbent assays (Millipore Corp., Bedford, USA).

### Hypothalamic, hippocampal and serum levels of 5-HT, 5-HIAA, and catecholamines

Three days after the infusion experiment and after an overnight fast, some animals were euthanized and their hypothalami and hippocampi were dissected and immediately weighed and homogenized in 1.5 ml of cold 0.1 M perchloric acid containing 0.02% sodium metabisulfite and 0.7 nM 3,4-dihydroxybenzylamine as internal standard (Watanabe et al., 2010). Levels of serotonin (5-HT) and 5-hydroxyindoleacetic acid (5-HIAA) were analyzed in 200 μl of serum. After centrifugation, the supernatant was stored at −80°C until analysis. The contents of 5-HT, 5-HIAA, and catecholamines were determined by High-pressure Liquid Chromatography (HPLC) with electrochemical detection.

### RNA extraction and real-time polymerase chain reaction (RT-PCR)

Four days after the behavioral tests, some animals were euthanized and their hypothalami and hippocampi were dissected and immediately stored at −80°C until analysis. Total RNA from hypothalami and hyppocampi were extracted using Trizol protocol (Invitrogen). One nanogram of total RNA, as determined by NanoDrop 1100 (NanoDrop Technologies, Wilmington, DE, USA), was used for cDNA synthesis (High-Capacity cDNA kit, Applied Biosystems). The cDNA was amplified using the TaqMan® Universal PCR Master Mix Kit with specific TaqMan Gene Expression. The following target genes were assessed: serotonin 1A (5-HT_1A_) receptor (Rn00561409_s1), serotonin 1B (5-HT_1B_) receptor (Rn01637747_s1), serotonin 2C (5-HT_2C_) receptor (Rn00562748_m1), serotonin trasporter (5-HTT) (Rn00564737_m1), neuropeptide Y (NPY) (Rn01410145_m1), pro-opiomelanocortin (POMC) (Rn00595020_m1), cocaine- and amphetamine-regulated transcript (CART) (Rn01645174_m1), agouti-related peptide (AgRP) (Rn01431703_g1), leptin receptor (ObR) (Rn 01433205_m1), adiponectin receptor 1 (AdipoR1) (Rn01483784_m1), and adiponectin receptor 2 (AdipoR2) (Rn01433173_m1). β-actin (Rn00667869_m1) was used as endogenous control for normalization. The PCR reactions were performed in a 96-well Optical Reaction Plate (Applied Biosystems, Foster City, CA, USA). The thermocycler parameters were as follows: 50°C for 2 min, 95°C for 10 min, 50 cycles of 95°C for 15 s and 60°C for 1 min. Expression of the target genes was normalized against that of the Sham animals. The statistical analysis was performed using the ΔCt value (Ct_gene of interest_ – Ct_β−actin_) and the results were expressed using the 2-ΔΔCt method (Livak and Schmittgen, 2001).

### Behavioral tests

#### Intracerebroventricular serotonin injection and food intake measurement

At the eighth week of treatment, the animals were anesthetized with ketamine/xylazine (66/33 mg/kg, ip) and stereotaxicaly implanted with a guide cannula (21 gauge, 15 mm length) aimed at the lateral cerebral ventricle (from bregma: −0.9 mm posterior, +1.6 mm lateral and −2.5 mm ventral) (Paxinos et al., 1985). They were then caged individually with free access to food and water during 1 week. After this recovery period, they were fasted for 6 h and received an intracerebroventricular (ICV) injection of either 5.0 μl of vehicle (artificial cerebrospinal fluid, CSF: 145 mmol/l sodium chloride, 2.7 mmol/l potassium chloride, 1.2 mmol/l calcium chloride, 2.0 mmol/l di-sodium hydrogen phosphate, and 1 mmol/l magnesium chloride at pH 7.4) or 5.0 μl of vehicle containing either 200 or 300 μg of serotonin (H9523, Sigma-Aldrich, USA). The injections were performed in the animal room immediately before lights off. Following the injection, they were returned to their individual cages and a known amount of diet was offered. Diet consumption was assessed by weighting the food remaining after 12 and 24 h. Each animal was injected twice, receiving vehicle or one serotonin dose, on separate days, two days apart. They were randomly divided so that half the animals received vehicle as the first injection and the other half received serotonin as the first injection. The correct cannula positioning was evaluated by the dipsogenic effect of an ICV 20 ng dose of angiotensin II. All animals failing to drink water were discarded.

#### Elevated plus maze test

The maze consisted of two open arms and two closed arms connected by a central platform high off the ground and lit by a dim light. The animals were placed individually on the central platform facing an open arm. The test was performed during 5 min and was videotaped for subsequent analysis. The time spent, number of entries and distance traveled in the arms were measured by two observers (Pellow et al., 1985). The number of entries and distance traveled in the closed arms were used as a measure of locomotor activity (Campos et al., 2013). The anxiety index was calculated by the equation: 1-[(open arm time/5 min) + (open arm entry/total entry)]/2. The index values range from 0 to 1, with a higher value indicating increased anxiety (Huynh et al., 2011).

#### Modified forced swim test

The rats were individually placed in a Plexiglas cylinder (diameter 30 cm, height 50 cm) containing water up to 30 cm (or high enough to prevent the animal from supporting the body with the tail) at 25 ± 1°C. On the first day, the animals remained in the cylinder for 15 min (training session). After 24 h, the procedure was repeated for 5 min (test session). The test session was videotaped for subsequent analysis. The predominant behavior within each 5-s period was recorded. The following behaviors were assessed: swimming (movements throughout the cylinder), climbing (upward-directed movements with the forepaws along the cylinder walls), immobility (floating with minimal movements with head just above the water) and number of dips (Porsolt et al., 1977). After the sessions, the animals were dried and returned to the home cage.

## Statistical analysis

Data are expressed as mean ± SEM. Comparisons among the groups (Sham, OvxC, OvxL, and OvxF) were performed by ANOVA followed by Tukey's test for multiple comparisons. The effect of intracerebroventricular serotonin on food intake was assessed in each group by Student's *t*-tests for dependent measures. The Pearson's correlation coefficient was used to analyze the existence of relationships between behavioral parameters in the elevated plus maze and forced swim tests, and metabolic/gene expression data. Multivariate regression analysis was performed to assess metabolic/gene expression factors influencing behavioral variables. The regression models were constructed based on the statistically significant correlations shown by the univariate analysis. The Statistics Software Package for the Social Sciences (SPSS, v18.0) was used for these analyses.

## Results

### Lard diet but not fish diet potentiated the deleterious effects of ovariectomy on body weight gain and metabolic/hormonal parameters

The daily food mass and food energy intakes of the control, lard and fish diets were measured once a week, throughout the 8 weeks, and the cumulative intakes are shown in Table 1. There was no effect of ovarietomy, either alone (OvxC group) or combined with high-fat feeding (OvxL and OvxF groups), on the cumulative food intake, while the cumulative caloric intake of the OvxL group was significantly higher than that of the OvxC group. These findings indicate that, although the two hyperlipidic diets had the same caloric content, only the lard diet induced increments of the caloric intake of the ovariectomized rats.

**Table 1:** Body and serum parameters of Sham, OvxC, OvxL, and OvxF groups.

**Variables**	**Groups**			
	**Sham**	**OvxC**	**OvxL**	**OvxF**
Cumulative daily food intake (g/8 weeks)	171.94 ± 14.27 (*n* = 19)	163.12 ± 5.71 (*n* = 12)	161.71 ± 2.81 (*n* = 17)	148.00 ± 4.94 (*n* = 20)
Cumulative daily caloric intake (kcal/8 weeks)	493.47 ± 40.95 (*n* = 19)	468.15 ± 16.40^&^ (*n* = 12)	582.16 ± 10.11^#^ (*n* = 17)	532.78 ± 17.78 (*n* = 20)
Feed efficiency (g/Kcal/8 weeks)	10.77 ± 0.86 (*n* = 19)	16.95 ± 0.78^*^^$^ (*n* = 12)	17.31 ± 0.80^*^^$^ (*n* = 17)	12.88 ± 0.82^#^^&^ (*n* = 18)
Body weight gain (g/8 weeks)	49.90 ± 1.96 (*n* = 20)	70.51 ± 3.04 (*n* = 16)	101.67 ± 3.67^*^^^#^^$^^ (*n* = 18)	70.18 ± 3.42 (*n* = 18)
Final body weight (g)	247.91 ± 3.80 (*n* = 20)	277.84 ± 4.98^*^ (*n* = 16)	307.16 ± 5.22^*^^^#^^$^^ (*n* = 18)	281.11 ± 3.86^*^ (*n* = 18)
Adipose depots (g/100g)	4.40 ± 0.24 (*n* = 14)	4.77 ± 0.25 (*n* = 8)	7.17 ± 0.27^*^^#^^$^ (*n* = 12)	4.12 ± 0.39^#^ (*n* = 9)
Glucose (mg/dl)	98.04 ± 2.38 (*n* = 9)	116.53 ± 3.8^*^ (*n* = 11)	128.67 ± 2.66^*^ (*n* = 13)	128.28 ± 5.52^*^ (*n* = 13)
Insulin (ng/ml)	1.08 ± 0.07 (*n* = 6)	1.19 ± 0.21 (*n* = 6)	1.87 ± 0.43^$^ (*n* = 6)	0.74 ± 0.17^&^ (*n* = 6)
Leptin (ng/ml)	2.09 ± 0.49 (*n* = 6)	4.30 ± 0.40 (*n* = 9)	9.21 ± 1.27^*^#^$^ (*n* = 10)	2.50 ± 0.28 (*n* = 9)
Triglycerides (mg/dl)	55.67 ± 7.45 (*n* = 7)	67.71 ± 1.81 (*n* = 8)	67.83 ± 7.21 (*n* = 10)	32.25 ± 2.30^*^^^#^^ (*n* = 8)
Total cholesterol (mg/dl)	63.32 ± 5.02 (*n* = 5)	79.77 ± 3.87 (*n* = 5)	90.16 ± 9.84^*^ (*n* = 5)	61.23 ± 2.39^&^ (*n* = 5)

Values are Mean ± SEM

* p < 0.05 vs. Sham;

# p < 0.05 vs. OvxC;

& p < 0.05 vs. OvxL;

$ *p < 0.05 vs. OvxF*.

Ovariectomy, either *per se* or combined with high-lard intake, increased feed efficiency while the high-fish intake prevented this effect of ovariectomy, indicating a protective effect of the high-fish diet. Ovariectomy increased body weight gain and final body weight. In the OvxL group, these parameters were higher than in the OvxC and OvxF groups. These results show that the excess weight gain induced by ovariectomy was deepened by the lard diet but not by the fish diet. Taken together, these data suggest that increased feed efficiency may explain the high body weight gain of the OvxC and OvxL groups but not of the OvxF group.

Body adiposity, estimated by the sum of 3 adipose tissue depots, was higher in the OvxL animals than in all the other groups [*F*_(3, 44)_ = 22.817, *p* = 7.55 × 10^−9^; *p* = 1.87 × 10^−7^ vs. Sham; *p* = 1.43 × 10^−4^ vs. OvxC; *p* = 1.66 × 10^−8^ vs. OvxF). Additionally, the OvxF group showed decreased adiposity in comparison to OvxC (*p* = 0.044). These data indicate that ovariectomy associated with lard consumption, but not with fish-oil consumption, caused obesity.

All the Ovx groups showed increased levels of serum glucose, regardless the diet consumed [*F*_(3, 45)_ = 11.288, *p* = 1.46 × 10^−5^]. Insulin levels were higher in OvxL than in OvxF [*F*_(1, 20)_ = 90,527, *p* = 7.25 × 10^−8^]. Although without statistical significance (*p* = 0.597), insulin levels of OvxF rats were 62% lower than those of OvxC animals. These data suggest that fish-oil intake ameliorated the insulin resistance induced by ovariectomy.

Leptinemia was increased in OvxL [*F*_(3, 33)_ = 16.839, *p* = 1.88 × 10^−6^] in relation to all the groups. The OvxF rats showed a nonsignificant (*p* = 0.511) reduction of leptin levels in relation to OvxC group. These results are in accordance with the adiposity data.

OvxF animals presented reduced triglyceride levels [*F*_(3, 32)_ = 9.023, *p* = 2.23 × 10^−4^] in relation to all the other groups. In addition, total cholesterol levels were higher in OvxL [*F*_(3, 20)_ = 5.817, *p* = 0.006] than in Sham (*p* = 0.024), and lower in OvxF than in OvxL (*p* = 0.014). The data indicate that ovariectomy affected blood glucose levels regardless the diet. However, in ovariectomized rats, the consumption of lard impaired while fish-oil intake presented a protective effect on metabolic and hormonal parameters.

### Fish diet stimulated hippocampal serotonergic activity in ovariectomized rats

Because monoamines participate in the control of both metabolic and behavioral aspects, their serum levels as well as their hypothalamic and hippocampal tissue levels were evaluated and are presented in Table 2.

**Table 2:** Catecholamines, serotonin and metabolites levels of the Sham OvxC, OvxL, and OvxF groups.

**Variables**	**Groups**			
	**Sham**	**OvxC**	**OvxL**	**OvxF**
**SERUM**				
5-HT (pg/ml)	607.81 ± 68.54	600.06 ± 25.32	558.73 ± 44.55	466.59 ± 34.95
5-HIAA (pg/ml)	10.38 ± 0.36	10.09 ± 1.32	8.33 ± 0.26	8.96 ± 1.06
**HYPOTHALAMUS**				
Noradrenaline (pg/g)	422.48 ± 26.06	419.45 ± 20.76	459.64 ± 8.98	412.70 ± 15.79
Adrenaline (pg/g)	12.17 ± 0.87	11.43 ± 0.59	14.21 ± 1.64	14.23 ± 1.59
L-Dopa (pg/g)	6.03 ± 1.22	6.20 ± 0.86	7.55 ± 0.88	6.036 ± 0.96
Dopamine (pg/g)	101.54 ± 10.29	102.85 ± 4.46	117.86 ± 11.14	110.50 ± 9.04
5-HIAA (pg/g)	290.64 ± 43.54	317.66 ± 18.24	365.95 ± 43.96	420.43 ± 33.00
5-HT (pg/g)	160.14 ± 18.29	192.93 ± 3.10	215.85 ± 23.11	180.17 ± 11.20
5-HIAA/5-HT	1.83 ± 0.20	1.66 ± 0.12	1.72 ± 0.11	2.36 ± 0.22
**HIPPOCAMPUS**				
Noradrenaline (pg/g)	56.04 ± 5.46	50.58 ± 4.37	47.58 ± 5.43	52.18 ± 2.25
Adrenaline (pg/g)	1.44 ± 0.15	1.46 ± 0.17	1.80 ± 0.38	1.68 ± 0.12
L-Dopa (pg/g)	0.95 ± 0.23	0.87 ± 015	1.27 ± 0.24	1.89 ± 0.23^*^^#^
Dopamine (pg/g)	3.30 ± 0.49	3.31 ± 0.63	3.40 ± 0.68	4.73 ± 1.04
5-HIAA (pg/g)	147.31 ± 12.13	163.32 ± 8.33	166.04 ± 9.15	187.27 ± 7.56^*^
5-HT (pg/g)	89.84 ± 9.37	106.79 ± 5.28	103.63 ± 4.91	87.61 ± 5.77
5-HIAA/5HT	1.69 ± 0.18	1.55 ± 0.10	1.60 ± 0.10	2.18 ± 0.21^#^

Values are mean ± SEM n = 5–6.

* p < 0.05 vs. Sham;

# *p < 0.05 vs. OvxC*.

Both circulating and hypothalamic levels of serotonin, catecholamines and their metabolites failed to present significant differences among the groups.

In the hippocampus, L-Dopa levels were higher [*F*_(3, 20)_ = 4.596, *p* = 0.016] in OvxF than in Sham (*p* = 0.036) and OvxC (*p* = 0.016) rats. 5-HIAA levels were higher in OvxF than in Sham (*p* = 0.044) while serotonin turnover was higher in OvxF [*F*_(3,19)_ = 3.535, *p* = 0.039] than in OvxC (*p* = 0.038). The present data indicate that the serotonergic activity was stimulated in the hippocampus of the OvxF animals.

### Ovariectomy plus high-lard feeding impairs hypothalamic serotonergic system gene expression

Hypothalamic and hippocampal mRNA levels of several genes related to metabolic and behavioral homeostasis were determined and are presented in Tables 3, 4.

**Table 3:** Hypothalamic gene expression of Sham, OvxC, OvxL, and OvxF animals.

**Genes**	**Groups**			
	**Sham**	**OvxC**	**OvxL**	**OvxF**
5-HT_1A_	1.01 ± 0.09	0.79 ± 0.09	0.68 ± 0.08	0.81 ± 0.03
5-HT_1B_	1.00 ± 0.03	0.94 ± 0.07	0.71 ± 0.03^*^	0.94 ± 0.06
5-HT_2C_	1.01 ± 0.10	1.55 ± 0.13^*^	1.26 ± 0.04	1.53 ± 0.12^*^
5-HTT	1.07 ± 0.15	0.98 ± 0.13	0.63 ± 0.11	1.06 ± 0.10
POMC	1.04 ± 0.19	1.41 ± 0.23	1.36 ± 0.10	1.02 ± 0.10
CART	1.03 ± 0.15	1.03 ± 0.11	1.19 ± 0.11	1.12 ± 0.09
NPY	1.00 ± 0.07	0.93 ± 0.05	0.89 ± 0.05	0.90 ± 0.06
AgRP	1.01 ± 0.11	0.76 ± 0.17	0.47 ± 0.04^*^	0.51 ± 0.02^*^
AdipoR1	1.00 ± 0.03	1.09 ± 0.06	1.04 ± 0.02	1.10 ± 0.07
AdipoR2	1.00 ± 0.01	0.86 ± 0.03	0.85 ± 0.05	0.79 ± 0.02^*^
ObR	1.00 ± 0.06	1.07 ± 0.04	0.81 ± 0.11	0.90 ± 0.01

*The mRNA levels were measured by RT-PCR and expression of the target genes was normalized against that of the Sham animals*.

Values are mean ± SEM n = 5–6. The housekeeping gene β-actin mRNA was used to normalize the relative amounts of mRNA.

* *p < 0.05 vs. Sham*.

**Table 4:** Hippocampal gene expression of Sham, OvxC, OvxL, and OvxF animals.

**Genes**	**Groups**			
	**Sham**	**OvxC**	**OvxL**	**OvxF**
5-HT_1A_	1.02 ± 0.12	0.96 ± 0.15	0.91 ± 0.09	0.80 ± 0.08
5-HT_1B_	1.04 ± 0.17	1.05 ± 0.06	1.16 ± 0.06	0.96 ± 0.10
5-HT_2C_	1.00 ± 0.04	0.98 ± 0.16	1.50 ± 0.27	1.35 ± 0.19
5-HTT	1.20 ± 0.40	0.79 ± 0.13	0.78 ± 0.18	0.76 ± 0.09
POMC	1.01 ± 0.10	1.43 ± 0.21	0.90 ± 0.09	1.36 ± 0.12
CART	1.07 ± 0.23	1.17 ± 0.21	1.15 ± 0.25	1.14 ± 0.22
NPY	1.00 ± 0.01	0.95 ± 0.09	1.09 ± 0.13	0.99 ± 0.06
AgRP	1.01 ± 0.10	0.88 ± 0.08	0.89 ± 0.09	1.12 ± 0.10
AdipoR1	1.00 ± 0.01	0.94 ± 0.02	1.09 ± 0.08	1.14 ± 0.11
AdipoR2	1.00 ± 0.04	0.83 ± 0.04	0.94 ± 0.10	0.77 ± 0.05
ObR	1.02 ± 0.12	1.06 ± 0.17	1.21 ± 0.20	1.02 ± 0.02

*The mRNA levels were measured by RT-PCR and expression of the target genes was normalized against that of the Sham animals*.

*Values are mean ± SEM n = 5–6. The housekeeping gene β-actin mRNA was used to normalize the relative amounts of mRNA*.

As shown in Table 3, OvxL had lower 5-HT_1B_ hypothalamic gene expression [*F*_(3,15)_ = 5.042, *p* = 0.017] than Sham (*p* = 0.017) while 5-HT_2C_ expression was higher in OvxC and OvxF (*p* = 0.018 and *p* = 0.021, respectively) when compared to the Sham group [*F*_(3, 15)_ = 5.767, *p* = 0.011]. AgRP expression was lower in OvxL and OvxF (*p* = 0.018 and *p* = 0.029, respectively) than in Sham rats [*F*_(3, 15)_ = 5.458, *p* = 0.013]. AdipoR2 gene expression [*F*_(3, 15)_ = 5.847, *p* = 0.011] was lower in OvxF than in Sham animals (*p* = 0.008).

These data show that the intake of lard or fish-oil by the ovariectomized rats affected distinctly the components of the hypothalamic serotonergic system. While the effect of the lard diet pointed to a deleterious consequence on the serotonergic effectiveness, both ovariectomy alone or combined with fish-oil intake tended to yield to an upregulation of serotonin action.

No significant changes were found in hippocampal gene expression among the groups (Table 4).

### Fish diet reverses the ovariectomy-induced impairment of serotonin hypophagia

**Figure 1:**
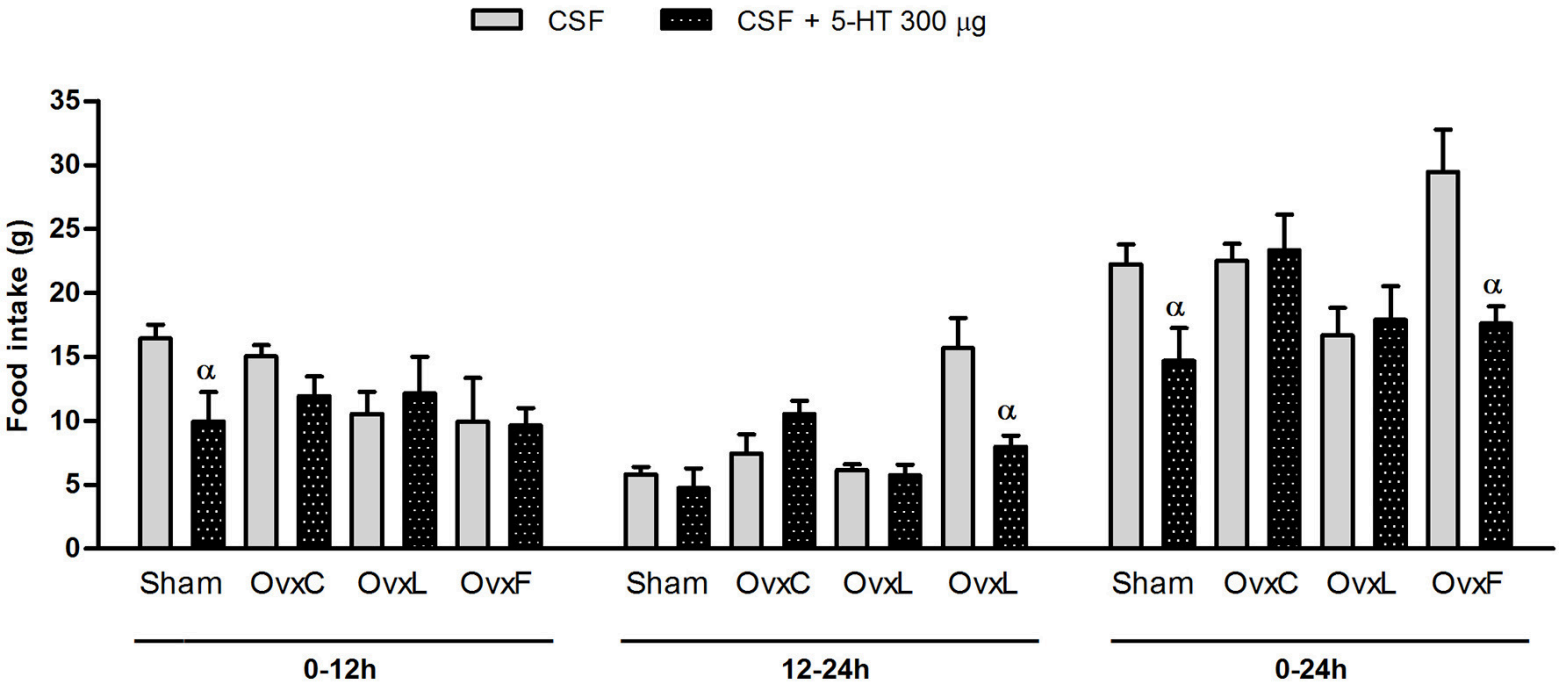
Food intake of Sham (*n* = 4–6), OvxC (*n* = 4–5), OvxL (*n* = 4–5) and OvxF (*n* = 4–5) groups during the first and the second 12-h periods and the total 24-h period following the intracerebroventricular injection of vehicle (solid bars) or 300 μg of serotonin (dotted bars). ^α^*p* < 0.05 vs. vehicle.

In order to analyze the hypophagic effect of intracerebroventricular serotonin after ovaries removal and chronic consumption of different high-fat diets, the animals received 200 or 300 μg of serotonin ICV. The results are shown in Figure 1.

The ICV injection of 200 μg of serotonin had no effect on food intake (data not shown). The dose of 300 μg of serotonin evoked a significant hypophagic effect on Sham animals in the first period of 12 h (*p* = 0.026) and in the cumulative period of 24 h (*p* = 0.025). In addition, OvxF presented a significant decrement of food intake in the second period of 12 h (*p* = 0.009) and in the cumulative period of 24 h (*p* = 0.007) after serotonin injection. Contrastingly, serotonin failed to significantly inhibit feeding in OvxC and OvxL animals.

These findings show that ovariectomy suppressed the ability of central serotonin to inhibit food intake. The use of the fish-oil diet, but not of the lard diet by the ovariectomized rats restored serotonin hypophagia.

### Either high-fat diet reversed the anxiogenic effect of ovariectomy

**Figure 2:**
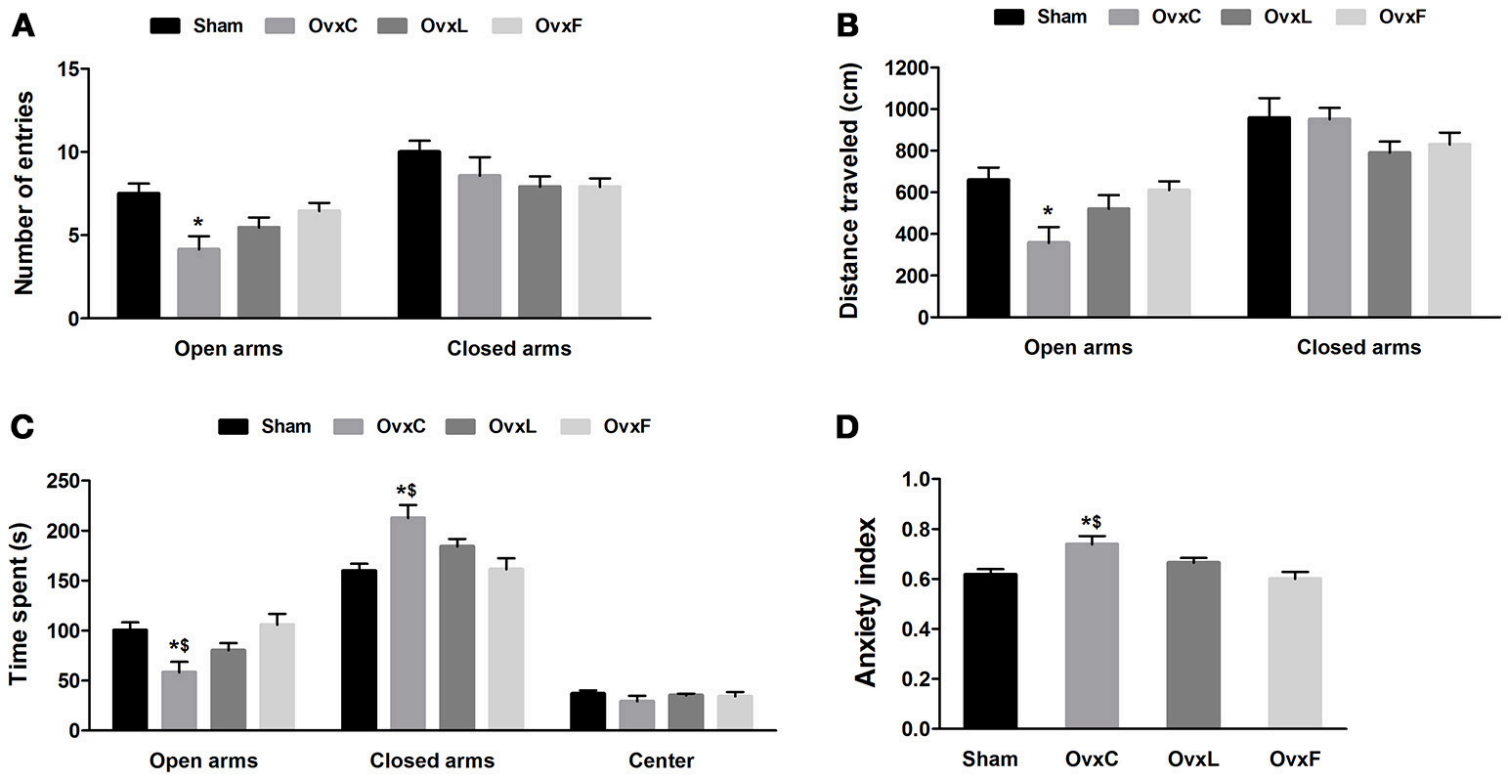
Number of entries **(A)**, distance **(B)**, and percentage of time spent **(C)** in the open or closed arms, and anxiety index **(D)** during the elevated plus maze test of Sham (*n* = 12), OvxC (*n* = 8), OvxL (*n* = 9), and OvxF (*n* = 11) rats. ^*^p < 0.05 vs. Sham; ^$^*p* < 0.05 vs. OvxF.

The anxiety-like behavior was assessed in the elevated plus maze test, which is based on the conflict between exploration of the environment and the fear of the potential danger resulting from this exposure (Pellow et al., 1985). As illustrated in Figure 2, the OvxC animals entered fewer times [*F*_(3, 38)_ = 4.843, *p* = 0.006] (Figure 2A) and traveled a shorter distance in the open arms [*F*_(3, 38)_ = 3.881, *p* = 0.017] (Figure 2B) than the Sham rats (*p* = 0.005 e *p* = 0.013, respectively). Moreover, OvxC also spent less time exploring the open arms [*F*_(3, 38)_ = 5.025, *p* = 0.005] and more time in the closed arms [*F*_(3, 38)_ = 6.063, *p* = 0.002] when compared to both Sham and OvxF groups (*p* = 0.003 and *p* = 0.006, respectively) (Figure 2C). There were no significant differences among the groups regarding the number of entries and the distance traveled in the closed arms (Figures 2A,B, respectively). As shown in Figure 2D, OvxC presented higher anxiety index [*F*_(3, 38)_ = 5.128, *p* = 0.05] when compared to Sham (*p* = 0.012) and OvxF (*p* = 0.006) animals.

These findings show that ovariectomy induced an anxiety-like behavior, evidenced by lower exploration of the open spaces of the maze. However, high-fat diets consumption, independent of the fatty acids type, reverted this anxiogenic effect of ovariectomy.

### Ovariectomy allied to high-lard intake induced depressive-like behavior

**Figure 3:**
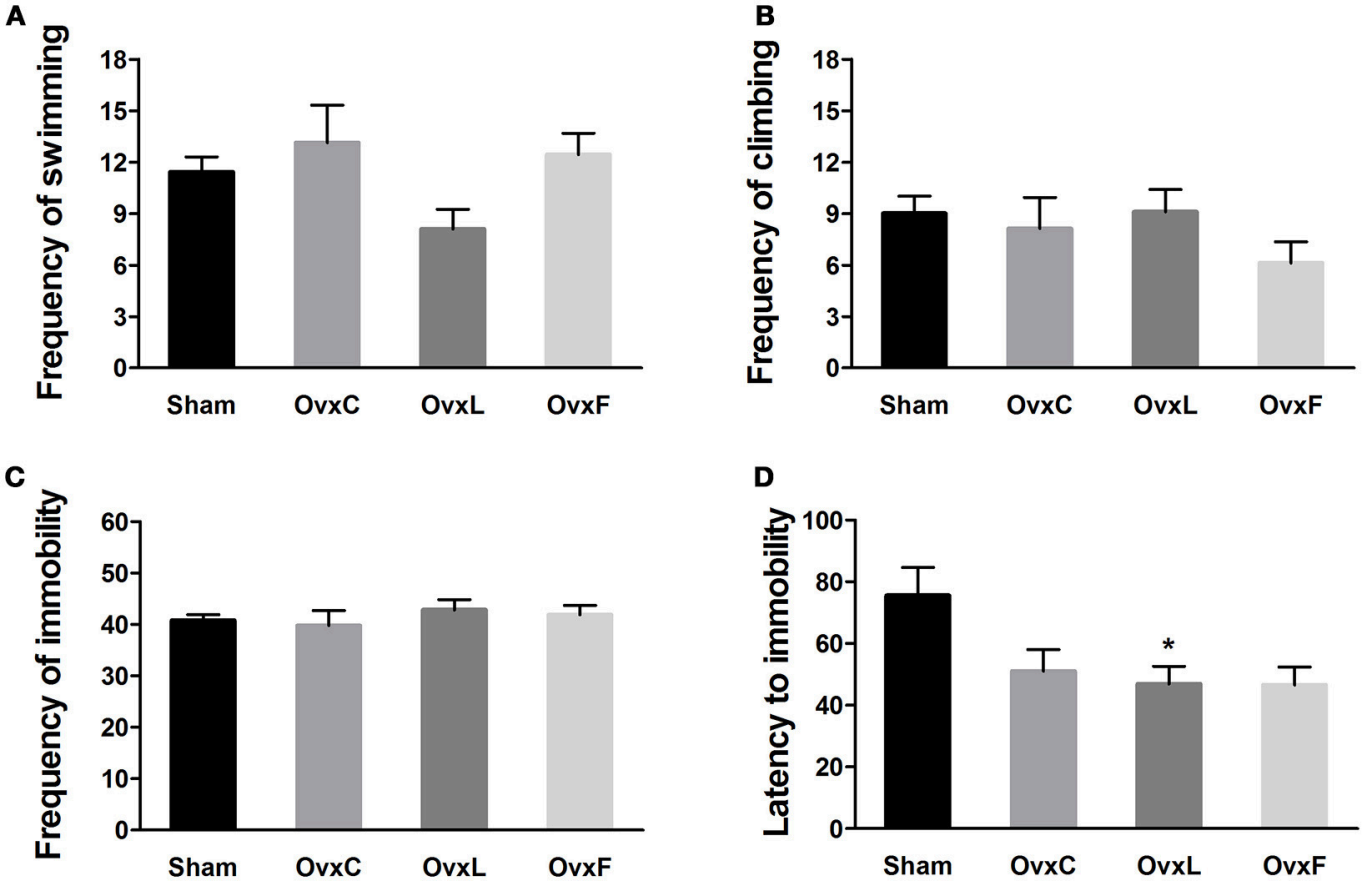
Swimming **(A)**, climbing **(B)**, immobility frequencies **(C)**, and latency to immobility **(D)** of Sham (*n* = 14), OvxC (*n* = 8), OvxL (*n* = 12), and OvxF (*n* = 9) rats during the forced swim test. ^*^p < 0.05 vs. Sham.

The depressive-like behavior was assessed by the forced swimming test, in which the frequency of immobility is considered the main indicative of depressive-like state (Porsolt et al., 1977). There were no significant differences in swimming (Figure 3A), climbing (Figure 3B), and immobility frequencies (Figure 3C) among the groups. However, the OvxL group showed a non-significant decrement of swimming frequency when compared to the Sham group (*p* = 0.528). As illustrated in Figure 3D, the latency to immobility was shorter in the OvxL group [*F*_(3, 41)_ = 3.920, *p* = 0.016] than in the Sham group (*p* = 0.028). These results indicate that ovariectomy allied to high-lard intake induced vulnerability to a depressive-like state.

### AgRp gene expression associations indicate beneficial effects on depression and anxiety measures

The correlation analyses were performed to determine the associations between behavioral and metabolic variables. The following parameters were included in the correlation analysis: all parameters of the elevated plus maze test and of the forced swimming test, body weight gain, adipose depots mass and their sum, and all genes expression data. Table 5 depicts the variables showing at least one significant correlation.

**Table 5:** Pearson's correlation coefficients of the Sham, OvxC, OvxL, and OvxF groups.

	**EOA**	**ECA**	**DOA**	**DCA**	**AI**	**Rearing**	**HD**	**FI**	**LI**	**FS**
BW gain	−0.33^β^	−0.27	−0.33^β^	−0.28	0.08	−0.24	−0.05	−0.04	−0.21	−0.23
Fat depots sum	−0.15	−0.05	−0.08	−0.11	0.25	−0.06	0.02	0.11	−0.24	−0.40^β^
**HIPPOCAMPUS**										
5-HT_1B_	−0.18	−0.01	−0.05	0.19	0.42	0.08	0.15	0.52^β^	−0.10	−0.32
5-HT_2C_	−0.20	0.02	−0.11	0.02	−0.11	−0.31	0.48	0.49	−0.32	−0.59^β^
SERT	−0.29	0.09	−0.23	0.19	0.37	−0.51^β^	0.03	0.34	0.07	−0.08
AgRP	0.36	0.14	0.28	0.00	−0.83^β^	0.06	0.48	−0.26	0.12	−0.01
POMC	−0.08	−0.07	−0.16	0.14	−0.18	0.00	−0.09	−0.30	−0.28	0.61^β^
AdipoR1	0.25	−0.03	0.28	−0.20	−0.23	−0.35	0.67^β^	0.05	−0.44	−0.28
AdipoR2	−0.04	0.55	−0.09	0.17	0.22	−0.48	−0.17	0.15	0.09	−0.24
**HYPOTHALAMUS**										
NPY	0.08	0.56^β^	0.06	0.60^β^	−0.25	−0.25	0.04	0.17	0.11	−0.03
AgRP	0.01	0.38	−0.12	0.15	0.00	−0.26	−0.28	−0.17	0.69^β^	0.11
AdipoR1	0.21	0.11	0.10	0.05	−0.25	−0.03	0.17	−0.41	−0.50^β^	0.41
AdipoR2	0.11	0.53^β^	−0.05	0.21	0.00	−0.40	−0.23	−0.10	0.45	−0.08

*Pearson's correlation coefficients between behavioral body adiposity and gene expression parameters (n = 15–16)*.

EOA, number of entries in open arms; ECA, number of entries in closed arms; DOA, distance traveled in open arms; DCA, distance traveled in closed arms; AI, anxiety index; HD, headdipping; FI, frequency of immobility; LI, latency of immobility; FS, frequency of swimming.

β *p < 0.05*.

In the elevated plus maze test, the number of entries in the open arms presented an inverse correlation with the total body weight gain (*p* = 0.040), while the number of entries in the closed arms showed direct associations with hypothalamic gene expression of NPY (*p* = 0.025) and AdipoR2 (*p* = 0.033). The distance traveled in the open arms had an inverse association with body weight gain (*p* = 0.045) while the distance traveled in the closed arms showed a direct correlation with NPY hypothalamic gene expression (*p* = 0.014). The anxiety index presented an inverse correlation with hippocampal AgRP expression (*p* = 0.004). Rearing behavior had a negative association to SERT expression in hippocampus (*p* = 0.042) while the number of headdippings was positively related to AdipoR1 gene in hippocampus (*p* = 0.005).

In the forced swimming test, the frequency of immobility presented a direct association with 5-HT_1B_ hippocampal gene expression (*p* = 0.038) while the latency to immobility had a positive association with hypothalamic AgRP (*p* = 0.004) and a negative association with hypothalamic AdipoR1 (*p* = 0.0.46). The frequency of swimming had an inverse correlation with the sum of adipose depots (*p* = 0.008) and with hippocampal 5-HT_2C_ (*p* = 0.017), and a direct association with hippocampal POMC gene (*p* = 0.012).

These results showed associations of hippocampal and hypothalamic gene expression rates with behavioral measures indicative of anxious-like and depressive-like behaviors. We then used linear regression models to identify predictors for the variations of the behavioral parameters anxiety index, frequency of immobility and latency to immobility, among the hypothalamic and hippocampal genes presenting significant correlation with these behavioral parameters (Table 6).

**Table 6:** Linear regression models for predictors of behavioral parameters.

**Predictors**	**Beta coefficients**	**Standard error**	***p*-value**
**DEPENDENT VARIABLE: ANXIETY INDEX**			
AgRP *hpc*	−0.34	0.06	8.11^−5^
Intercept	0.95	0.06	3.72^−10^
**DEPENDENT VARIABLE: FREQUENCY OF IMMOBILITY**			
5-HT_1B_ *hpc*	11.46	4.99	0.037
Intercept	30.80	5.38	5.27^−5^
**DEPENDENT VARIABLE: LATENCY TO IMMOBILITY**			
AgRP *hpt*	58.81	17.15	0.004
Intercept	11.97	12.06	0.33

*hpc, hippocampus; hpt, hypothalamus*.

For the anxiety index, the model predicted 68% of the variations [*R* = 0.85, *R*^2^ = 0.68, *F*_(1, 14)_ = 30.024, *p* < 0.00008] and showed that hippocampal AgRP (*p* = 3.01^−5^) was a negative predictor (power 0.9959, effect size 2.1250, 2 predictors). The other predictor tested was the hippocampal 5-HT_1B_ serotonin receptor but its effect was not significant.

The linear regression model for frequency to immobility in the forced swimming test predicted 27% of the variations [*R* = 0.52, *R*^2^ = 0.27, *F*_(1, 14)_ = 5.270, *p* = 0.037) and showed that hippocampal 5-HT_1B_ gene expression was a positive predictor (*p* = 0.037) (power 0.4474, effect size 0.3698, 2 predictors). The other predictor tested was the hippocampal 5-HT_2C_ serotonin receptor but its effect was not significant.

For the latency to immobility, the linear regression model predicted 47% of the variations [*R* = 0.68, *R*^2^ = 0.47 *F*_(1, 13)_ = 11.749, *p* = 0.004] and showed that hypothalamic gene expression of AgRP (*p* = 0.004) was a positive predictor (power 0.8279, effect size 0.8867, 2 predictors). The other predictor tested was the hypothalamic AdipoR1 but its effect was not significant.

Overall, the linear regression models pointed to AgRp gene expression associations indicative of a beneficial involvement of this neuropeptide on both depression and anxiety measures.

## Discussion

To investigate mechanisms involved in the putative interaction of metabolic disturbances and mood and anxiety disorders developed after menopause, we examined the effects of ovariectomy alone or in combination with chronic consumption of high-fat diets on metabolic/neurochemical and behavioral parameters of rats. The diets differed in their fat source by the use of either lard (rich in saturated fatty acids) or fish-oil (rich in n-3 polyunsaturated fatty acids).

Ovariectomy induced excess body weight gain in the absence of excess food intake, probably as result of increased feed efficiency. Additionally, since previous studies in animals and humans have reported basal metabolism decrements in estrogen-deficient states (Ainslie et al., 2001; Monda et al., 2006; Witte et al., 2010), this aspect, not evaluated in the present study, may have played a role.

The intake of the lard diet exacerbated the body weight gain of the ovariectomized group and increased body adiposity and leptinemia. The saturated fatty acids have the highest obesogenic ability (Buettner et al., 2006), a fact associated with its higher rate of acylation into triglycerides and lower rate of oxidation in comparison to polyunsaturated and monounsaturated fatty acids (Siddiqi et al., 2000). Chronic intake of lard-enriched diet impaired carnitine-palmitoyl-transferase-1 function, damaging the mitochondrial import and oxidation of long chain fatty acids (Noland et al., 2009).

In contrast, although similar in caloric density, the fish-oil diet reduced body adiposity, tryglicerides and total cholesterol levels, and restored feed efficiency to levels similar to those of the Sham rats. Numerous studies have shown that n-3 polyunsaturated fatty acids present beneficial metabolic effects (Misra et al., 2010; Abeywardena and Patten, 2011; Robinson and Mazurak, 2013). In relation to their effect on adipose tissue, omega-3 fatty acids reportedly modulated gene expression through transcription factors such as peroxisome proliferator-activated receptor γ (PPARγ), resulting in the stimulus of fatty acids oxidation in mitochondria and peroxisomes (Hensler et al., 2011). Docosahexaenoic acid (DHA, 22:6n-3) and eicosapentaenoic acid (EPA, 20:5n-3) presented hypolipidemic effects, in part due to inhibition of fat cell proliferation (Ruzickova et al., 2004). Additionally, omega-3 fatty acids stimulated the generation of new mitochondria in adipocytes and activated the expression of the carnitin-palmitoyl-transferase 1 gene (Flachs and Hal, 2005).

In the present study, all ovariectomized groups presented hyperglycemia, in agreement with the disrupted glycemic control induced by estrogen deficiency (Ross and Polotsky, 2012). Inadequate glycemic control has been directly associated with depression in humans (Lustman et al., 2000). This agrees with the present findings that, although only lard intake allied to ovariectomy caused a statistically significant decrease of the latency to immobility (a measure indicative of increased propensity to develop depression), this parameter showed a relevant decrement in all ovariectomized groups.

As expected, the intracerebroventricular administration of serotonin induced hypophagia in the Sham animals. In contrast, this effect was completely abolished in the OvxC and OvxL groups, while it was preserved in the OvxF group. Serotonin induces hypophagia by modulating the expression of the hypothalamic orexigenic and anorexigenic neuropeptides (Choi et al., 2003, 2006; Ronan and Summers, 2011; Donovan and Tecott, 2013). Serotonin binding to 5-HT_1B_ reportedly inhibited NPY/AgRP orexigenic neurons, while its binding to 5-HT_2C_ activated anorexigenic POMC/CART neurons (Heisler et al., 1999, 2006; Garfield and Heisler, 2009). The present results of hypothalamic gene expression showed increased expression of 5-HT_2C_ in the OvxC and OvxF animals. In view of the lack of serotonin hypophagia in OvxC group, it can be suggested that 5-HT_2C_ upregulation may have represented a compensatory mechanism, aimed at surpassing receptor insensitivity. It is interesting that the high-fat diets affected differentially serotonin receptor expressions. The lard diet had a deleterious effect, both abolishing 5-HT_2C_ upregulation and inducing 5-HT_1B_ downregulation. These effects are highly consistent with the absence of serotonin hypophagia after lard intake. In contrast, although fish-oil intake induced receptor expression alterations similar to those seen in the OvxC group, serotonin hypophagia was maintained. The factors leading to the absence of hypophagia in OvxC, but not in OvxF, are not apparent, as 5-HT_2C_ expression was elevated in both groups. One contributing factor may derive from our previous data, in male rats, showing that fish-oil intake increased neuronal activation of hypothalamic sites expressing anorexigenic mediators, indicating a positive effect of fish oil in mobilizing multiple hypothalamic anorexigenic pathways (Watanabe et al., 2009).

The present findings showing that both metabolic and behavioral alterations were caused by ovariectomy agree with the reported homeostatic role of ovarian hormones (Humeniuk et al., 2011; Kiss et al., 2012; Messina et al., 2013). Additionally, the present demonstration of significant associations between neuropeptides and serotonin receptors with behavioral parameters highlight the existence of a complex connection between them.

The intake of either the lard or the fish-oil diet normalized the anxiety index of the Ovx animals, indicating an anxiolytic effect of the high-fat diets, regardless the fatty acid composition. A previous report showed that myristic acid (C16:0) produced anxiolytic-like effects comparable to those of diazepam in male rats (Contreras et al., 2014). We have previously reported myristic acid as the most abundant fatty acid in the lard-enriched diet consumed by our animals (Dornellas et al., 2015). It has been shown that palatable foods, such as diets rich in sugar and lard, reduced signs of stress and anxiety in humans and animals (Finger et al., 2011; Singh, 2014). Furthermore, OvxF rats had higher hippocampal concentrations of L-Dopa and 5-HIAA and elevated serotonin turnover when compared to the Sham rats. In accordance, it has been reported that supplementation with fish oil reversed the reduced hippocampal serotonin level in mice under chronic mild stress (Vancassel et al., 2008), indicating an anxiolytic effect of fish oil, probably mediated by stimulated serotonergic activity.

Serotonergic neurons play an important role in mood disorders and serotonin deficiency is known to be a substantial contributing factor in anxiety and depression (Brigitta, 2002; Leonardo and Hen, 2006; Kormos and Gaszner, 2013). The present data showed hippocampal 5-HT_1B_ gene to be a positive predictor of the frequency of immobility during the forced swimming test, a paradigm of depressive-like behavior in rats. The 5-HT_1B_ receptor inhibits serotonin release acting as an auto-receptor in serotonergic neurons and as a hetero-receptor in hippocampal gabaergic neurons (Clark et al., 2006; López-Pantoja et al., 2012). Mice lacking 5-HT_1B_ auto-receptors displayed increased extracellular serotonin levels in the ventral hippocampus, following the administration of a selective serotonin reuptake inhibitor, and decreased anxiety-like behavior and antidepressant-like effects in the forced swim and sucrose preference tests (Nautiyal et al., 2016). Additionally, estrogen replacement induced an antidepressant-like action in ovariectomized rats, but this effect did not occur in animals with destruction of serotonergic innervation (Vega-Rivera et al., 2013). These data show that 5-HT_1B_ activity decreases serotonergic activity and support the present finding of this receptor as a positive predictor of depression, as observed in OvxL animals.

The present experiments indicated the possible involvement AgRP neuropeptide in modulating both anxious- and depressive-like behaviors. The linear regression model found AgRP gene expression in hippocampus to be a negative predictor of the anxiety index. Additionally, hypothalamic AgRP expression was a positive predictor of the latency to immobility. The latter finding is in agreement with the present observation of decreased hypothalamic AgRP and latency to immobility by ovariectomy allied to high-fat saturated diet. Little is known about the influence of neuropeptides regulating energy homeostasis on emotional behaviors. Importantly, reduction of AgRP signaling increased extracellular dopamine levels in the basal forebrain, leading to increased exploratory behavior and response to cocaine (Dietrich et al., 2012). Moreover, brain AgRP infusion reduced locomotor activity (Tang-Christensen et al., 2004). The present results add relevant information to the understanding of the influence of hypothalamic neuropeptides on nonfood-associated behaviors, indicating a possible involvement of AgRP on emotional disruption mechanisms leading to the anxiety and depression states observed after menopause. Further experiments are required to verify the role played by AgRP as a modulator of anxious- and depressive-like behaviors.

The present findings contributed to the characterization of the metabolic, neurochemical and behavioral changes induced by ovarian failure and demonstrated the effects of diet manipulations, highlighting a positive effect of high-fat feeding on the anxiety-like behavior shown by ovariectomized animals. Since the polyunsaturated omega-3 intake (fish diet), unlike the saturated fat intake (lard diet), failed to induce deleterious metabolic or neurochemical consequences, further studies are needed focusing on the potential of this dietary component as an adjuvant anxiolytic agent after menopause.

## Author contributions

AD conception and design, interpretation of data; drafting and revising it critically; final approval of the work, in agreement to be accountable for all aspects of the study. VB conceptualization, design, execution, and interpretation of the research study. LC conceptualization, execution, and interpretation of the research study. IdA conceptualization, design, execution, and or interpretation of the research study. AP conceptualization and interpretation of the research study. TV-F design, execution, and interpretation of the research study. CdS execution, and interpretation of the research study. LO revising the study critically for important intellectual content, final approval of the version to be published. CdN revising the study critically for important intellectual content, final approval of the version to be published. ER conception and design, interpretation of data; drafting and revising it critically; final approval of the work, in agreement to be accountable for all aspects of the study.

### Conflict of interest statement

The authors declare that the research was conducted in the absence of any commercial or financial relationships that could be construed as a potential conflict of interest.
