# Corrigendum: High-Fat Feeding Improves Anxiety-Type Behavior Induced by Ovariectomy in Rats

**DOI:** 10.3389/fnins.2018.00738

**Published:** 2018-10-12

**Authors:** Ana P. S. Dornellas, Valter T. Boldarine, Amanda P. Pedroso, Lorenza O. T. Carvalho, Iracema S. de Andrade, Tânia M. Vulcani-Freitas, Carla C. C. dos Santos, Cláudia M. da Penha Oller do Nascimento, Lila M. Oyama, Eliane B. Ribeiro

**Affiliations:** Physiology Department, Escola Paulista de Medicina, Universidade Federal de São Paulo, São Paulo, Brazil

**Keywords:** hypothalamus, hippocampus, serotonin, lard, fish-oil, neuropeptides

In the original article, we neglected to include the funder Fundação de Amparo à Pesquisa do Estado de São Paulo-FAPESP, Grants No. 2012/03172-4 and the funder Conselho Nacional de Desenvolvimento Científico e Tecnológico-CNPq, Grants No. 309405/2013-0 and 453924/2014-0. The authors apologize for this error and state that this does not change the scientific conclusions of the article in any way.

The original article has been updated.

## Conflict of interest statement

The authors declare that the research was conducted in the absence of any commercial or financial relationships that could be construed as a potential conflict of interest.

